# *ALK*融合基因阳性非小细胞肺癌的研究进展

**DOI:** 10.3779/j.issn.1009-3419.2018.09.10

**Published:** 2018-09-20

**Authors:** 鹏 宋, 力 张, 聪聪 尚

**Affiliations:** 100730 北京，中国医学科学院 北京协和医学院北京协和医院呼吸内科 Department of Respiratory Medicine, Peking Union Medical College Hospital, Chinese Academy of Medical Science & Peking Union Medical College, Beijing 100730, China

**Keywords:** ALK融合基因, NSCLC, ALK抑制剂, 耐药, 检测方法, *ALK* gene, NSCLC, ALK-TKI, Drug resistance, Detection method

## Abstract

*ALK*基因重排在非小细胞肺癌（non-small cell lung cancer, NSCLC）中发生率约3%-5%。*ALK*基因抑制剂近年来取得极大突破，明显延长了ALK阳新晚期NSCLC患者的生存期；但大部分患者持续用药后会出现获得性耐药。本文分别从*ALK*基因背景、检测方法、三代ALK抑制剂的治疗效果以及耐药后的策略、展望进行了阐述。希望对临床工作有参考价值和借鉴意义。

## Abstract (en)

The incidence of *ALK* gene rearrangement in non-small cell lung cancer (NSCLC) was about 3% to 5%. *ALK* gene inhibitors have made great breakthrough in recent years, significantly extending the survival period of patients with ALK(+) advanced NSCLC. But the majority of patients will be acquired drug resistance after treatment. This article has been explained separately from the ALK genetic background, the detection method, the treatment of the three generations of ALK inhibitors and the strategy after drug resistance. It is desire to have reference value and reference meaning for clinical work.

肺癌的发病率及致死率在恶性肿瘤中均占首位。根据最新发表在*CA: A Cancer Journal for Clinicians*杂志的Cancer statistics，2017数据显示，2017年美国新发肺癌病例约11万人，死亡约7万人^[1]^。约85%的为非小细胞肺癌（non-small cell lung cancer, NSCLC），此类患者就诊时多为晚期，既往含铂两药方案1年生存率不足40%^[2]^。随着肿瘤信号转导通路研究的发展，分子靶向治疗受到越来越多的关注，其中约5%的NSCLC患者体内存在间变淋巴瘤激酶（naplastic lymphoma kinase, *ALK*）基因融合，以ALK酪氨酸激酶区与5’末端棘皮动物微管结合蛋白（echinoderm microtubule-associated protein-like 4, EML4）形成融合基因融合最为常见^[3]^。美国每年约确认ALK阳性患者1万人^[4]^。本文就*ALK*融合基因阳性NSCLC的最新进展进行综述。

## 1 *ALK*融合基因的研究背景

*ALK*基因于1994年在间变性大细胞淋巴瘤（ALCL）中被发现，ALCL中存在的*NPM-ALK*融合基因具备致癌特性^[5]^。后续多项研究发现炎性肌纤维母细胞瘤、神经母细胞瘤等均与ALK基因突变相关^[6]^。2007年日本学者Soda M等^[7]^通过蛋白组学技术在肺腺癌肿瘤组织中首次发现*ALK*基因突变：*EML4*基因的1-13号外显子与*ALK*基因的20-29号外显子融合形成*EML4-ALK*融合基因。融合基因的EML4（尤其是Basic区）具有强大的致癌活性，这种活性主要依靠EML4-ALK通过二聚化激活酪氨酸激酶，从而活化下游的JAK/STAT、PL3K/mToR及MAPK等多条通路导致细胞增殖与凋亡失控。转染了*EML4-ALK*融合基因的NIH-3T3成纤维细胞具备了无限增殖的能力^[8]^。

截至目前，至少发现了15种EMLK-ALK融合变体亚型，其中，最常见的是EML4的变体1（v1：外显子13与ALK的外显子20融合[E13; A20]）和变体3：（v3a/b：外显子6a/b与ALK的外显子20融合[E6a/b; A20]）（图 1）。两种发生率超过60%。所有的变体都保留了ALK的整个酪氨酸激酶结构域和EML4的N末端卷曲螺旋区域，这对于ALK的二聚化和组成型激活是必不可少^[9]^。除EML4这一最常见的融合伴侣外，多项研究发现TFG、KLC1、SOCS5、H1P1、TPR、BIRC 6等多种少见的ALK融合伴侣，它们的相关临床研究罕见^[10-12]^。期待相关数据的出现。

**1:**
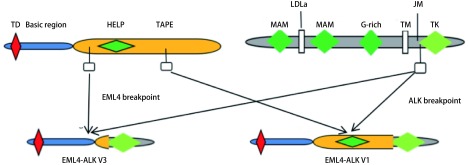
*EML4*与*ALK*融合基因常见变体示意图 The schematic diagram of *EML4* and *ALK* fusion gene common variants

## 2 *ALK*融合基因检测方法新进展

现在有4种不同方法来检测*ALK*融合基因的表达，分别是逆转录聚合酶链反应（reverse transcription-polymerase chain reaction, RT-PCR）、免疫组化（immunohistochemistry, IHC）、荧光原位杂交法（fluorescence *in situ* hybridization, FISH）及第二代测序技术（next-generation sequencing, NGS）。FISH法是美国国家综合癌症网（National Comprehensive Cancer Network, NCCN）推荐的*ALK*融合基因检测金标准。它利用互补DNA探针的3’和5’端，然后用荧光显微镜进行观察*ALK*基因所在的2p23区域。Vysis ALK FISH探针试剂盒已被FDA批准用于ALK重排的检测，但是FISH荧光信号的快速消减及价格昂贵限制了其应用^[13]^。RT-PCR技术在实验室中应用更为广泛，但其需要以ALK融合伴侣进行引物设计，石蜡组织标本RNA可能保存不当出现假阳性，且组织用量大，使RT-PCR技术的临床应用受到诸多限制^[14]^。相对而言，IHC有高效、快速及价格低廉的特点，因而可广泛用于常规的病理实验室，近期由罗氏公司推出的首款全自动VENTANA ALK（D5F3）IHC检测试剂盒已获CFDA及FDA认证，该法检测融合基因的特异性与敏感性分别为98%与100%^[15]^。但IHC的结果判定存在很大主观性，很大程度上依赖于抗体的质量。2017年11月16日基于二代测序技术（NGS）的癌症基因检测分析平台MSK-IMPACT^TM^被食品药品监督管理局（Food and Drug Administration, FDA）批准，该平台可以1次对肿瘤的468个基因的基因突变进行快速、灵敏的检测，可对这些基因上所有重要区域进行测序，并能检测基因上所有的拷贝数变化、启动子突变、基因组重排及蛋白编码区突变。该技术不仅可准确检测*ALK*融合基因，更能准确检测罕见突变及其他的遗传变异，毋庸置疑，该技术将为ALK阳性NSCLC的初始治疗及耐药后治疗策略提供重要参考^[16]^。

最近Letovanec I等依据欧洲大型胸科肿瘤平台Lungscape队列中切除NSCLC的标本对所有四种技术检测ALK突变的吻合率及一致性进行了探讨，对60个样本同时使用四种方法检测，其中55个样本的检测结果是一致的（43个ALK+，12个ALK-）。如果以IHC和FISH两种技术检测的一致性结果作为标准，则RT-PCR和NGS检测的灵敏度是70%和85%，检测的特异性是87.1%和79%。如果将IHC与RT-PCR或NGS结合起来，其灵敏度没有提示，但是检测的特异性提升至了88.7%和83.9%。因此，为了达到最高的灵敏度和特异性，*ALK*突变应用两种技术进行检测，而第三种方法则可在以上结果不一致时进行评估^[17]^。

## 3 ALK抑制剂的研究进展（图 2）

**2:**
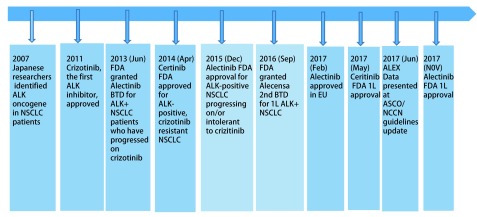
ALK抑制剂的发展历程 The development history of ALK-TKI

众所周知，EML4-ALK代表了一种NSCLC的分子亚型，多见于不吸烟、年轻女性、腺癌及EGFR野生型患者。因EML4-ALK阳性者的某些特征与*EGFR*突变者相似，2011年ASCO年会韩国的Kim等对1, 100例2003年-2009年期间非鳞Ⅲb期-Ⅳ期NSCLC患者的临床资料进行了分析，研究者将患者分为三组：ALK阳性、*EGFR*突变型、ALK和EGFR均野生型。结果显示，三组患者一线接受含铂方案化疗的PFS无差异，但接受EGFR-TKI治疗的ALK阳性患者，PFS率低于*EGFR*突变组（*P* < 0.001）和ALK/EGFR野生型组（*P*=0.048）。因此EML4-ALK阳性者不能从EGFR-TKI靶向治疗中获益，且接受EGFR-TKI治疗的野生型患者和ALK阳性患者相比后者可能更容易产生耐药。多种ALK激酶抑制剂应运而生。

### 3.1 第一代ALK抑制剂—克唑替尼（Crizotinib）

PROFILE 1001是关于Crizotinib的首个Ⅰ期临床试验，共149例NSCLC患者入组，客观缓解率（objective response rate, ORR）为60.8%，其中有3例达到完全缓解（complete response, CR）。中位无进展生存期（median progression-free survival, mPFS）为9.7个月，1年生存率为74.8%^[18]^。PROFILE 1005为多中心、单臂Ⅱ期临床试验，在可统计的259例患者中，ORR为60%，mPFS为8个月。随后的一项国际多中心、随机、开放Ⅲ期临床研究PROFILE 1007，比较了Crizotinib与化疗（培美曲塞或多西他赛）的疗效与安全性，两组ORR分别为65%和20%，mPFS为7.7个月和3.0个月^[19]^。而另一项Ⅲ期临床研究PROFILE 1014比较了克唑替尼和含铂两药方案对未经治疗的NSCLC的治疗效果。共招募343例患者进入，两组mPFS为10.9个月和7.0个月，两组ORR分别为74%和45%^[20]^。在2017年欧洲肿瘤医学会（European Society for Medical Oncology, ESMO）大会上，报道了PROFILE 1014再随访36个月后OS的数据，两组中位OS分别为未达到（NR）和47.5个月，校正后的统计分析结果显示，克唑替尼组的OS数据显著改善，有可能超过5年^[21]^。针对东亚人群的PROFILE 1029研究，得出两组的mPFS为11.1个月和6.8个月，ORR分别为88%与46%，显示亚裔人群使用克唑替尼一线治疗获益更多^[22]^。基于以上研究结果克唑替尼作为ALK阳性NSCLC患者一线治疗的地位被奠定。

克唑替尼相关不良反应轻微，主要为腹泻、恶心及呕吐等1级-2级胃肠道不良反应及视力障碍，而中性粒细胞减少及转氨酶升高等3级-4级不良反应也比较常见。但大约2.5%的病人在克唑替尼治疗后出现间质性肺疾病，需引起临床医生的警惕。

### 3.2 第二代ALK抑制剂——Ceritinib

ASCEND-1是首次关于Ceritinib的Ⅰ期临床试验，共招募59例ALK阳性的NSCLC患者，确定最大耐受计量为750 mg/d，后纳入71例患者，这130例患者ORR为58%，其中80例此前接受过克唑替尼治疗^[23]^。ASCEND-2是1项多中心、单臂Ⅱ期临床试验，共纳入140名经1线-3线化疗后进展的ALK阳性NSCLC患者，其中71.4%有脑转移，该研究ORR为38.6%（脑转移患者ORR为33%），颅内ORR为45%。mPFS为5.7个月（脑转移病例mPFS为5.4个月，无脑转移病例mPFS为11.3个月）^[24]^。基于以上结果，2014年4月29日FDA批准Ceritinib用于治疗ALK阳性经克唑替尼治疗后进展或不能耐受的转移性NSCLC（图 1）。

ASCEND-4是1项关于Ceritinib对比传统化疗的Ⅲ期临床试验，其研究结果在2016年WCLC大会上公布。本研究共纳入未经任何治疗的376例患者，经商化独立中心（BIRC）分析，Ceritinib组与化疗组的mPFS为16.6个月与8.1个月（HR=0.55），两组的ORR分别为26.7%与72.5%，中位缓解持续时间（DOR）为23.9个月*vs* 11.1个月。对于存在基线脑转移的患者，Ceritinib组颅内ORR为72.7%，化疗组颅内ORR为27.3%，mPFS为10.7个月与6.6个月（HR=0.70）^[25]^。ASCEND-5对比了Ceritinib与化疗治疗既往化疗和克唑替尼治疗进展后的患者，Ceritinib组与化疗组mPFS分别为5.4个月*vs* 1.6个月^[26]^。2017年5月26日，基于ASCEND-4的结果，FDA快速批准了Ceritinib用于ALK阳性初治NSCLC的一线适应症。目前尚未有Ceritinib头对头对比克唑替尼相关的数据。

Ceritinib的不良反应以腹泻、恶心、呕吐及肝酶升高为主，总体安全可控。2017年WCLC大会上，Byoung chal等报道了ASCEND-8的结果，发现450 mg/d Ceritinib的15个月无进展生存率估计值较750 mg/d Ceritinib高，这为临床不能耐受Ceritinib副作用提供了一种有效的剂量调整方案^[27]^。

### 3.3 第二代ALK抑制剂——Alectinib

AF-001JP是Alectinib的1项Ⅰ期/Ⅱ期研究，主要纳入未经ALK抑制剂治疗的患者。Ⅰ期试验建议300 mg bid为临床研究阶段推荐剂量。Ⅱ期试验ORR为93.5%。24个月无进展生存率为76%，24个月总生存率达79%^[28]^。2017年世界肺癌大会（World Conference on Lung Cancer, WCLC）上，Makoto Nishio等公布了AF-001JP长期随访结果, PFS超越4年。AF-002JG是Alectinib的另一项Ⅰ期/Ⅱ期研究，纳入47例既往使用过ALK-TKI不耐受或进展患者，ORR为55%（1例达CR）。21例有基线脑转移的患者，ORR为50%，疾病控制率（disease control rate, DCR）为90%，5例达CR^[29]^。NP28761和NP28763是两项关于Alectinib的Ⅱ期研究，纳入对克唑替尼耐药的ALK阳性NSCLC，两项研究的ORR为47.8%与49.2%。肿瘤缩小病例对Alectinib持续应答的中位时间为7.5个月与11.0个月。有基线脑转移的患者，ORR为66.8%和55.9%，mPFS为6.3个月与8.9个月。基于以上结果，FDA于2015.12.11批准Alectinib用于ALK阳性的二线治疗^[30]^。

2016年美国临床肿瘤学会（American Society of Clinical Oncology, ASCO）大会上，Alectinib对比克唑替尼初治ALK阳性NSCLC患者的研究结果公布（J-ALEX研究）。共招募207例患者，BIRC评估的两组ORR为91.6%与78.9%，mPFS为NR[20.3-NR]与10.2个月[8.2-12.0]（HR=0.34, *P*＜0.000, 1）。2017年ASCO大会公布了J-ALEX再随访10个月后的结果，Alectinib组与克唑替尼组相比mPFS为25.9个月与10.2个月，ORR为85%与70%^[31]^。并同时公布了ALEX研究（比较Alectinib和克唑替尼用于ALK阳性NSCLC一线治疗的疗效与安全性）的主要结果，共纳入303例Ⅲb期-Ⅳ期初治患者，BIRC评估mPFS为25.7个月*vs* 10.4个月（HR=0.50, *P*＜0.000, 1），首发中枢神经系统进展比例为Alectinib组12% *vs*克唑替尼组45%。两组的12个月颅内进展率为9.4%和41.4%（HR=0.16, *P*＜0.000, 1）。Alectinib组CNS缓解率和CNS DOR均明显提高，尚未有成熟的OS数据披露^[32]^。基于ALEX结果，FDA于2017年11月7日批准Alectinib用于一线治疗ALK阳性NSCLC。Alectinib是首个在头对头Ⅲ期研究中证实优于另一种TKI的靶向治疗药物，也是目前用于一线治疗mPFS最长的药物，Ⅲ期亚太研究已完成入组，期待早日在国内上市。

根据ALEX研究结果，Alectinib组3级-4级不良事件发生率为32%，克唑替尼组为56.7%，常见的不良反应为疲倦、便秘、水肿及鼻咽炎等，肝酶升高及重度肺炎等严重不良反应少见，表明Alectinib安全性显著优于克唑替尼及Ceritinib。

### 3.4 第二代ALK抑制剂—Brigatinib（AP26113）

Brigatinib是目前唯一的ALK、EGFR双靶点抑制剂，对ALK的抑制作用是克唑替尼的12倍。尤其对ALK G1202R突变具有作用，少见的氢键受体赋予其高度选择性的药效。

NCT01449461是关于Brigatinib的多中心Ⅰ期研究，纳入患者中包括79例ALK阳性的NSCLC，通过剂量递增确定Ⅱ期研究的剂量为180 mg qd。统计此前接受过克唑替尼耐药的患者，ORR为72%，颅内反应率为53%，平均无进展生存期为13.4个月，长于ceritinib和alectinib的同期数据^[33]^。ALTA Ⅱ期临床试验结果在2017年WCLC大会上公布，本研究分为A组（90 mg/d）和B组（90 mg/d共7天，转为180 mg/d）。分别经过16.8个月与18.6个月的中位随访期，IRC评估的两组ORR分别为51%与55%，DOR为13.8个月与14.8个月，mPFS为9.2个月与16.7个月；两组中位OS为NR和27.6个月。脑转移方面，两组基线脑转移可测量者颅内缓解率为50%与67%，DOR为NR与16.6个月；两组脑转移者（无论是是否为可测量病灶）mPFS为12.8个月与18.4个月，ALTA试验的疗效与安全性数据支持继续采用180 mg/d的给药方案进行Ⅲ期试验^[34]^。2017年4月28日基于该结果FDA批准Brigatinib用于二线治疗ALK阳性NSCLC。ALTA-1L研究（Brigatinib对比克唑替尼Ⅲ期研究）已经启动并于2016年4月开始招募患者，期待这一研究可为一线治疗再添有力武器。

Brigatinib总体安全性较好，最常见不良反应包括血肌酐及磷酸激酶升高、高血压、脂肪酶升高、肺炎及皮疹等，大多在减量或停药后好转。

### 3.5 第二代ALK抑制剂——Ensartinib（X-396）

Ensartinib是美国Xcovery公司研究的ALK、c-MET、ROS-1、ABL、SLK的多靶点抑制剂，体外实验证实ALK抑制活性是克唑替尼的3倍-10倍。对L1196M与C1156Y两个克唑替尼耐药靶点敏感，可有效克服克唑替尼的耐药性。

Ensartinib的Ⅰ期临床数据在2014年ASCO大会上公布。入组的30例患者有13例子为ALK阳性的NSCLC。常见的不良反应为恶心、水肿、乏力、呕吐及皮疹等，所有18例可评价实体瘤总有效率为56%，8例可评价肺癌中，剂量＞200 mg的6例患者总有效率为83%，中位治疗时间达20周，最长已达58周。推荐Ⅱ期临床剂量为225 mg/d。2016年ASCO大会报道了Ensartinib部分Ⅱ期临床数据，未经克唑替尼治疗的8例患者有效率为88%，PFS最长已超过32个月，进展患者为c-MET扩增；克唑替尼耐药者ORR为77%，PFS最长为28个月，3例进展患者为T1151M、L1196M及QSLP1188P+R1133Q+S1206F突变^[35]^。2017年欧洲肺癌大会（European Lung Cancer Congress, ELCC）大会公布了Ensartinib治疗脑转移的疗效，13例具有基线靶病灶的患者ORR为69%，DCR为10%；13例基线有非靶病灶的患者，中位缓解时间为5.8个月，最长达24个月^[36]^。

基于以上研究结果，Ensartinib头对头对比克唑替尼的Ⅲ期exalt3研究在2017年ASCO大会上公布了研究设计，中国有25家中心加入该研究，期待数据的早日公布。

### 3.6 第三代ALK抑制剂-Lorlatinib

Lorlatinib是辉瑞公司研发的1种强效ATP-竞争性ALK与ROS-1双重抑制剂，为唯一的三代ALK抑制剂。对已知的所有耐药突变均有效（L1198F突变除外）。2017年4月28日，FDA授予Lorlatinib治疗既往接受ALK抑制剂治疗进展ALK阳性NSCLC突破性药物资格。

2017年ASCO大会，Shaw等人公布了Lorlatinib治疗既往接受过≥1次ALK抑制剂的NSCLC有效性与安全性：Ⅰ期/Ⅱ期研究的结果。对既往接受过ALK-TKI治疗后耐药（包括G1202R）患者，Lorlatinib有较好疗效。ORR为46%，颅内ORR为42%，mPFS约9.6个月^[37]^。2017年第18届WCLC大会公布了Lorlatinib对伴有脑转移的ALK或ROS-1的NSCLC Ⅱ期研究结果，本研究纳入275例已接受或未接受治疗的脑转移NSCLC患者，根据治疗情况分队列进行分析：①未接受治疗的ALK+患者：ORR为90%（27/30），IC-ORR为75%（6/8）。②接受克唑替尼±化疗的ALK+患者：ORR为69%（41/59），IC-ORR为68%（25/37）。③接受克唑替尼之外ALK抑制剂±化疗的ALK+患者：ORR为33%（9/27），IC-ORR为42%（5/12）。④接受2-3种ALK抑制剂±化疗ALK+患者：ORR为39%（43/111），IC-ORR为48%（40/83）。表明Lorlatinib有强大临床意义的颅内活性，且与既往治疗线数无关^[38]^。Lorlatinib对比克唑替尼一线治疗ALK阳性NSCLC Ⅲ期研究CROWN（NTC03052608）已启动并开始招募患者。

Lorlatinib安全可控，常见不良反应为高脂血症与水肿，其余不良反应有周围神经病变，情绪认知影响，肝酶升高等；减量或停药可恢复。若出现罕见不良反应需永久停药。

## 4 ALK抑制剂的耐药机制（图 3）

**3:**
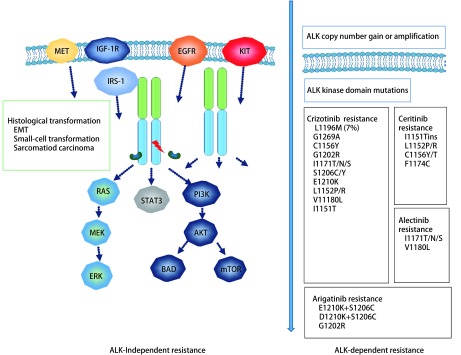
常见ALK抑制剂的耐药机制 Common ALK-TKI resistance mechanism

### 4.1 克唑替尼的耐药机制

#### 4.1.1 原发性耐药

目前针对克唑替尼原发性耐药的研究较少，一般定义用药3个月内进展的患者即视为原发性耐药，2017年ASCO大会广东省人民医院报道了克唑替尼原发性耐药的研究结果。通过对171例患者进行分析，发现18例患者存在原发性耐药，主要机制为非常见的ALK融合伴侣（ZC3H8-ALK，ALK-LOC102723854及ALK-DTNB-ASXL2）、BIM缺失多态性、PTEN/mTOR突变、ALK G3709A突变及KIT突变的联合等。原发性耐药患者的mPFS较获得性耐药患者明显缩短(2.2个月*vs* 10.8个月, *P* < 0.001）^[39]^。

#### 4.1.2 继发性耐药

克唑替尼继发性耐药突变约占克唑替尼耐药性问题的37%，主要包括ALK激酶区突变和*ALK*基因拷贝数的扩增，即所谓的ALK通路占优势耐药。

ALK激酶区突变的耐药机制比较明确，突变类型包括：G1269A、F1174L、L1152R、S1206Y、I152Tins、P1203N、V1180L、C1156Y、F1164V、G1202R、G1269S、L1196M，其中G1269A及L1196M最常见^[40, 41]^。最近有报道^[42]^ E1202K可能也是早期药物抵抗的1个点突变。患者发生克唑替尼耐药后，ALK点突变的类型可能有1种或上述几种的组合。

#### 4.1.3 ALK通路不占优势耐药

驱动基因的转换：ALK阳性NSCLC主要依赖ALK及其下游信号通路抑制肿瘤细胞的分裂与转移，当克唑替尼阻断该通路后，由于信号无法向下游传导，肿瘤细胞即可激活其他致癌驱动程序及其信号通路来代替ALK通路，即所谓的ALK通路占优势耐药。图 3主要包括*EGFR*突变、*KRAS*突变、KIT扩增、ErbB、MET扩增及下游信号通路IGF-1R等^[43]^。在细胞系研究中发现，EGFR-TKI抑制EGFR磷酸化可以恢复克唑替尼的敏感性，但尚不清楚ALK-TKI与EGFR-TKI的联合是否可提供更好的疗效及阻止ALK-TKI的耐药。现已有经克唑替尼治疗NSCLC转变为小细胞癌的报道^[44]^。

#### 4.1.4 肿瘤的异质性

NSCLC是基因及细胞异质性最强的肿瘤之一，随着分子生物学的进展，目前NSCLC的组织病理、分子遗传及单细胞均有了较高的认识，但NSCLC的真实面貌仍远超目前的认知水平。有研究认为，NSCLC在不同的时间及空间中，可能存在不同的驱动基因。小标本的基因检测是否可代表肿瘤组织全貌目前有待商榷。肿瘤复杂异质性的存在使耐药难以避免，期待早日对肿瘤异质性的起源做出解释。

### 4.2 下一代ALK抑制剂的耐药机制

克唑替尼耐药后使用二代ALK抑制剂可取得较好效果，但耐药仍无法避免。G1202R与F1174L是Ceritinib最常见的耐药突变^[45]^，C1156Y、1151Tins及L1152R等次级突变也被认为与Ceritinib的耐药性相关^[46]^。上皮细胞向间充质细胞的转化与E-钙黏连蛋白的减少、vimentin表达的增加有关^[47]^，MEK与SRC信号通路的激活^[48]^，这可能都是Ceritinib的潜在耐药机制。Alectinib常见的点突变包括I1171和V1180L^[42]^，肝细胞生长因子/MET旁路的激活是潜在的Alectinib耐药机制^[49]^。L1198F是最近报道的Lorlatinib耐药机制^[50]^，Brigatinin、X-396、Entrectinib因尚处于临床研究阶段，尚未有具体耐药机制的阐述。

### 4.3 EML4-ALK变体亚型对耐药的影响

2018年1月发表在JCO杂志上的1篇文章对127例患者ALK变体亚型与耐药机制的关系进行了研究，结果显示，V3患者在耐药后更容易出现ALK耐药突变，尤其是ALK G1202R突变；V3患者接受Lorlatinib的疗效劣于V1患者，但目前样本量小，期待后续的研究数据进一步证实。这一研究提示，有必要明确ALK阳性患者的变体亚型，可能有助于指导患者后续的治疗选择^[51]^。

## 5 展望未来的发展方向

下一代的ALK抑制剂正如雨后春笋般的出现，极大丰富了ALK(+)NSCLC的用药选择。继克唑替尼之后，ceritinib和alectinib都已进入一线治疗序列。但这些药物都没有成熟的OS数据，故目前关于ALK-TKI的一线药物选择上没有明确定论，也不清楚特定的ALK-TKI序列是否会影响肿瘤的生物学特性及特定的耐药机制。目前有两种主流观点：一种是一线使用PFS最长的药物，目前只有alectinib交出了满意答卷，其PFS最长可达24个月-26个月。另一种认为如果治疗初期就用最好的药物，产生耐药之后将面临无药可用的尴尬境地，2017年ESMO大会上法国学者报道了关于ALK抑制剂治疗时间的单中心研究，发现中位OS可达7年，他们认为最好策略是：先用克唑替尼，后续再根据耐药情况选择不同药物（表 1）。因此需要进行一项针对OS而不是PFS的临床试验来确定最佳的药物治疗序列，这可为临床选择ALK-TKI提供持久获益。

**1:** 5种ALK抑制剂的耐药和敏感突变(S:敏感，R:耐药，U:不清楚) Resistant and sensitive mutation of 5 ALK inhibitors (S: sensitive, R: resistant, U: unclear)

	Crizotinib	Alectinib	Ceritinib	Brigatinib	Lorlatinib
L1196M	R	S	S	S	S
G1269A	R	S	S	S	S
C1156Y	R	S	R	S	S
F1174L/C/V	R	S	R	S	S
1152Tins	R	S	R	S	S
L1152R	R	S	R	S	S
S1206Y	R	S	S	S	S
I1171L/C/V	R	R	S	U	S
G1202R	R	R	R	S	S
V1180L	R	R	S	U	S
G1123S	S	S	R	U	U
L1198F	S	R	R	R	R

另外，除了研发下一代更有效的ALK抑制剂克服耐药之外，ALT-TKI与旁路TKI或PD-1/PD-L1联合可能是克服耐药的新举措。期待相关临床试验的出现。
